# Identification of genetic modifiers enhancing B7-H3-targeting CAR T cell therapy against glioblastoma through large-scale CRISPRi screening

**DOI:** 10.1186/s13046-024-03027-6

**Published:** 2024-04-01

**Authors:** Xing Li, Shiyu Sun, Wansong Zhang, Ziwei Liang, Yitong Fang, Tianhu Sun, Yong Wan, Xingcong Ma, Shuqun Zhang, Yang Xu, Ruilin Tian

**Affiliations:** 1 School of Medicine, Southern University of Science and Technology, Shenzhen, Guangdong Province 518055 China; 2 Key University Laboratory of Metabolism and Health of Guangdong, Southern University of Science and Technology, Shenzhen, Guangdong Province 518055 China; 3 Department of Neurosurgery, Shenzhen People’s Hospital, Shenzhen, Guangdong 518020 China; 4 Department of Oncology, The Second Affiliated Hospital of Xi’an Jiaotong University, Xi’an, Shaanxi Province 710004 China

**Keywords:** Glioblastoma multiforme, CRISPR screening, CAR T cell, TNFSF15, B7-H3

## Abstract

**Background:**

Glioblastoma multiforme (GBM) is a highly aggressive brain tumor with a poor prognosis. Current treatment options are limited and often ineffective. CAR T cell therapy has shown success in treating hematologic malignancies, and there is growing interest in its potential application in solid tumors, including GBM. However, current CAR T therapy lacks clinical efficacy against GBM due to tumor-related resistance mechanisms and CAR T cell deficiencies. Therefore, there is a need to improve CAR T cell therapy efficacy in GBM.

**Methods:**

We conducted large-scale CRISPR interference (CRISPRi) screens in GBM cell line U87 MG cells co-cultured with B7-H3 targeting CAR T cells to identify genetic modifiers that can enhance CAR T cell-mediated tumor killing. Flow cytometry-based tumor killing assay and CAR T cell activation assay were performed to validate screening hits. Bioinformatic analyses on bulk and single-cell RNA sequencing data and the TCGA database were employed to elucidate the mechanism underlying enhanced CAR T efficacy upon knocking down the selected screening hits in U87 MG cells.

**Results:**

We established B7-H3 as a targetable antigen for CAR T therapy in GBM. Through large-scale CRISPRi screening, we discovered genetic modifiers in GBM cells, including *ARPC4*, *PI4KA*, *ATP6V1A*, *UBA1*, and *NDUFV1*, that regulated the efficacy of CAR T cell-mediated tumor killing. Furthermore, we discovered that TNFSF15 was upregulated in both *ARPC4* and *NDUFV1* knockdown GBM cells and revealed an immunostimulatory role of TNFSF15 in modulating tumor-CAR T interaction to enhance CAR T cell efficacy.

**Conclusions:**

Our study highlights the power of CRISPR-based genetic screening in investigating tumor-CAR T interaction and identifies potential druggable targets in tumor cells that confer resistance to CAR T cell killing. Furthermore, we devised targeted strategies that synergize with CAR T therapy against GBM. These findings shed light on the development of novel combinatorial strategies for effective immunotherapy of GBM and other solid tumors.

**Supplementary Information:**

The online version contains supplementary material available at 10.1186/s13046-024-03027-6.

## Background

Glioblastoma multiforme (GBM) is the most aggressive and common primary brain tumor, characterized by rapid growth, diffuse infiltration, and poor prognosis. With a median survival period of only 12–15 months and a 5-year survival rate of less than 5%, GBM poses a significant challenge in neuro-oncology [1–4]. The current standard therapy for GBM involves a combination of surgical resection, radiation therapy, and chemotherapy with temozolomide. However, the efficacy of these treatments is limited by the invasive nature of GBM cells, the presence of treatment-resistant tumor subpopulations, and the challenges posed by the blood-brain barrier [5]. Additionally, specific genetic alterations, such as *MGMT* promoter methylation, *EGFR* amplification, *PTEN* deletion, *IDH* mutations and *TERT* promoter mutations, also contribute to therapy resistance in GBM [6, 7]. Therefore, there is an urgent need to develop novel therapeutic modalities that can effectively target and eliminate GBM cells and overcome their inherent resistance in order to improve patient outcomes and ultimately find a cure for this devastating disease.

Chimeric Antigen Receptor T (CAR T) cell therapy has emerged as a revolutionary immunotherapy approach for cancer treatment. It involves modifying a patient’s own T cells to express a synthetic receptor (CAR), enabling them to recognize and target specific cancer cells [8]. CAR T therapy has demonstrated remarkable success in hematologic malignancies, such as leukemia and lymphoma [9, 10]. This success has spurred a growing interest in investigating the potential application of CAR T therapy in the treatment of solid tumors, including GBM [11]. Multiple targets have been explored so far for CAR T therapy against GBM, such as IL-13Rα2 [12–14], EGFR/EGFRvIII [15–17], EphA2 [18], HER2 [19, 20], B7-H3 (CD276) [21], GD2 [22], CD70 [23], CD133 [24], CD317 [25] and p32 [26]. Unlike chemotherapy drugs, which often face challenges in penetrating brain tumors due to the blood-brain barrier, intravenous infusion of CAR T cells has been shown to effectively cross the blood-brain barrier and enter the tumor in the brain [27, 28]. A few clinical trials have provided evidence of the feasibility, safety, and initial signs of efficacy of CAR T cell therapy in treating GBM [11]. However, the application of CAR T cells in GBM, like in other solid tumors, faces several limitations. GBM exhibits a high degree of antigen expression heterogeneity, thus highlighting the need to identify better targets with broader coverage and better safety profile [29, 30]. In addition, tumor cells develop resistance mechanisms that hinder the activation and effector functions of T cells. While mechanisms such as the PD-L1/PD-1 immune checkpoint axis are common to most tumor cells, many tumor-type specific resistance mechanisms remain to be uncovered [31, 32].

High-throughput genetic screening is a powerful approach for identifying cancer therapeutic targets. This process has been greatly facilitated by the recent development of CRISPR/Cas-based genetic manipulation tools, including CRISPR knockout (CRISPRn), CRISPR interference (CRISPRi) and CRISPR activation (CRISPRa) [33–35]. CRISPR-based genetic screening has been used in GBM recently to reveal mechanisms of resistance and uncover sensitizing targets for chemotherapy [36–38], radiotherapy [39, 40] and immunotherapy [41–43].

In the present study, we first established B7-H3 as a targetable antigen for CAR T therapy in GBM. Next, we uncovered genetic modifiers in GBM cells that modulated the efficacy of CAR T cell-mediated tumor killing through large-scale CRISPRi screening. Finally, we demonstrated an immunostimulatory role of TNFSF15 in modulating tumor-CAR T interaction to enhance CAR T cell anti-tumor activity.

## Materials and methods

### Cell culture

U87 MG, U251 MG, T98G and HEK293T cell lines were purchased from ATCC and cultured in Dulbecco’s Modified Eagle’s Medium (Gibco, Cat. no. C11995500BT) supplemented with 10% fetal bovine serum (TransGen Biotech, Cat. no. FS301-02) and 1% penicillin and streptomycin (Aladdin, Cat. no. P301861-100 ml) at 37 ℃and 5% CO_2_. All cell lines were mycoplasma negative detected by MycAway™ Plus-Color One-Step Mycoplasma Detection Kit (Yeasen, Cat. no. 40612ES25).

### Lentivirus production

For lentivirus production of the H1 library, 5 × 10^6^ HEK293T cells were seeded in a 15-cm dish for 24 h before transfection. 15 µg of H1 library plasmid and 15 µg of third-generation packaging mix (1:1:1 mix of the three plasmids) were diluted in 3mL of Opti-MEM (Gibco, Cat. no.31986-07). Subsequently, 120 µL of Polyethylenimine Linear (PEI) MW40000 (Yeasen, Cat. no. 40816ES03) was added to the 3 mL DNA dilution, vortexed for 10 s, and thoroughly mixed. After incubating at room temperature for 10–15 min, the mixture was added to a 15-cm dish containing HEK293T cells. Forty-eight hours later, the viral supernatants were collected and filtered with a 0.45 μm filter (Millipore, Cat. no. SLHV033RB).

For small-scale lentivirus production, 0.5 × 10^6^ HEK293T cells were seeded on 6-well plates. After 24 h, 100 µL of Opti-MEM was used for each well of cells to dilute 1 µg of transfer plasmid and 1ug of third-generation packaging mix, thoroughly mixed to form the DNA dilution. Subsequently, 4 µL of PEI was added, and the mixture was thoroughly vortexed for homogeneity. The remaining procedures were carried out as described above.

### Retroviral construct and retrovirus production

The scFv fragment targeting human B7-H3 (clone 376.96) was cloned into a previously validated CAR format that contains the hinge and transmembrane domains of human CD8α and the endo-domains of human CD28 and CD3ζ. The B7-H3.CAR cassette was cloned into the SFG-based bicistronic retroviral vector containing IRES and a mCherry reporter.

Retroviral supernatants used to transduce human T cells were prepared based on the described protocol [44]. Briefly, 3-3.5 × 10^6^ HEK293T cells were seeded in 10 cm cell culture dish and transfected with the plasmid mixture of the retroviral transfer vector, the PegPam plasmid encoding MoMLV gag-pol, and the RDF plasmid encoding the RD114 envelope, using the TransIT®-LT1 Transfection Reagent (Mirus, Cat. no. MIR2306), according to the manufacturer’s instruction. The supernatant containing the retrovirus was collected 48 and 72 h after transfection and filtered with 0.45 μm filters.

### Transduction and expansion of CAR T cells

Frozen peripheral blood mononuclear cells (PBMCs) from healthy donors were purchased from a commercial source (Stemcell). After thawing, PBMCs were activated on plates coated with 1 µg/mL CD3 (Miltenyi Biotec, Cat. no.130-093-387) and 1 µg/mL CD28 (BD Biosciences, Cat. no. 555,725) agonistic mAbs. On day 2, T lymphocytes were transduced with retroviral supernatants using retronectin-coated plates (Takara Bio Inc., Shiga, Japan, Cat. no. T100B). Three days post transduction, T cells are harvested and cultured in complete T cell media medium (X-VIVO™ 15 (Lonza, Cat. no. 04-418Q), 5% FBS (Hyclone, Cat. no.SV30208.02), 2 mM GlutaMAX, 100 unit/mL of Penicillin and 100 µg/mL of streptomycin) supplemented with IL-7 (10 ng/mL; PeproTech, Cat. no. 200-07-500) and IL-15 (5 ng/mL; PeproTech, Cat. no. 200-15-500), changing medium every 2–3 days [45]. On day 10–12 post transduction, T cells were collected and cultured in IL-7/IL-15 depleted T cell medium for one day prior and subsequently used for functional assays.

### Generation of CRISPRi-U87 cell line

U87 MG cells were co-transfected with plasmids encoding pC13N-dCas9-BFP-KRAB [46] and TALENS targeting the human CLYBL intragenic safe harbor locus (pZT-C13-R1 and pZT-C13-L1, Addgene #62,196 and #62,197) using Lipofectamine 3000 (Thermo Fisher Scientific, Cat. no. L3000001). Transduced cells were enriched based on the blue fluorescent protein (BFP) signal by fluorescence-activated cell sorting (FACS) using FACSAria SORP (BD Biosciences). We named this cell line CRISPRi-U87.

### CRISPRi screening

The workflow of the CRISPRi screen is illustrated in Fig. 3D. The H1 library which contains 13,025 unique sgRNA sequences targeting 2,318 kinases, phosphatases and drug targets (5 or 10 sgRNAs each gene), along with 500 non-targeting control sgRNAs, was packaged into lentivirus and transducted into CRISPRi-U87 at a low multiplicity of infection (MOI) of 0.3.

Then, the transduced cells were selected with 2 µg/mL of puromycin for 48 h to eliminate uninfected cells and generate a genome-edited cell pool. After selection, cells were expanded and divided into four groups, each containing 5 million cells. The cells were co-cultured with B7-H3 targeting or CD19 targeting CAR T cells derived from two healthy donors at an effector-to-target ratio of 1:4 for 36 h, followed by a 48-hour recovery period. Survived cells were then harvested, and the different cell populations were processed for next-generation sequencing to determine sgRNA abundancies in each group. Genomic DNA was extracted from CRISPRi-U87 cells with DNAiso Reagent (Takara, Cat. no. 9770Q) according to the manufacturer’s protocol. The sgRNA fragment was amplified using 2 × Phanta Flash Master Mix (Vazyme, Cat. no. P510-02) and size-selected using Hieff NGS DNA Selection Beads (Yeasen, Cat. no. 12601ES08). The sgRNA products were sequenced using a DNBSEQ-T7 instrument (MGI Tech). The MAGeCK-iNC pipeline was used for screening data analysis [46–48].

### sgRNA cloning

Individual sgRNAs were cloned into the pLG15 vector via BstXI and Bpu1102I sites as previously described [49]. The pLG15 vector contains a mouse U6 promoter-driven sgRNA expression cassette and an EF-1α promoter-driven puromycin resistance marker and BFP expression cassette for selection. A complete list of sgRNA sequences used in this study is listed in Supplementary Table 5.

### Co-culture experiments

Tumor cells were seeded in 12-well plates at 1 × 10^5^ cells/well. To assess the cytotoxic functions of CAR T cells, T cells were added to the culture at different CAR T (Effector) to tumor cell (Target) (effector-to-target, E: T) ratios (1:1, 1:2, or 1:4) without the addition of exogenous cytokines. Cells were analyzed on days 2–5 to measure residual tumor cells and T cells by FACS. Dead cells were gated out by Zombie Aqua Dye (Biolegend, Cat. no. 423,102) staining, while T cells were identified by the expression of mCherry and tumor cells by the expression of GFP (U87 MG, U251 MG, T98G cell lines and sgRNA-knocked down U87 MG sublines).

To detect cytokine production and activation markers of CAR T cells, a 5:1 E: T ratio was used, and culture supernatant and cells were collected 24 h post co-culture.

### ELISA

Cytokines (TNF-α, IFN-γ and IL-2) released by CAR T cells were measured in duplicate using specific ELISA kits (R&D system, Cat. no. DY202-05, DY285B-05, DY210-05) following manufacturer’s instructions. An 8-point dilution standard curve was performed for each ELISA plate.

### Flow cytometry

For surface staining, cells were incubated with antibodies at room temperature for 15 min or at 4 °C for 30 min. For staining of B7-H3 specific scFv molecules on the T cell surface, CAR T cells were incubated with recombinant B7-H3-Fc protein (Genscript) followed by Alexa Fluor 647-conjugated anti-Fc antibody (Biolegend, Clone M1310G05, Cat. no. 410,714).

For intracellular staining, cells were fixed and permeabilized using Cytofix/CytoPerm (BD Biosciences, Cat. no. 554,714) for 30 min at room temperature and washed with 1X PermWash (BD Biosciences, Cat. no. 554,714). Subsequent staining was performed using 1X PermWash as staining and wash buffer. In most assays, cells were stained with Zombie Aqua Live/Dead Viability dye (Biolegend, Cat. no. 423,102) to gate out dead cells for analysis.

The following antibodies used for the flow cytometry analysis were obtained from Biolegend: PE/Dazzle 594-conjugated anti-CD3 (Clone OKT3, Cat. no. 317,346), BV711-conjugated anti-CD4 (Clone OKT4, Cat. no. 317,440), Alexa Fluor 700-conjugated anti-CD8 (Clone SK1, Cat. no. 344,724), APC-conjugated anti-CD25 (Clone BC96, Cat. no. 302,610), BV650-conjugated anti-CD137 (Clone 4B4-1, Cat. no. 309,828), PE-conjugated anti-CD45RA (Clone H100, Cat. no. 304,108).

The following antibodies used for the flow cytometry analysis were obtained from BD Biosciences: FITC-conjugated anti-CCR7 (Clone 150,503, Cat. no. 561,271), APC-Cy7-conjugated anti-CD69 (Clone FN50, Cat. no. 557,756), V450-conjugated anti-Granzyme B (Clone GB11, Cat. no. 561,155).

Flow cytometry data were collected on NovoCyte Quanteon (Agilent) using NovoExpress software, and the flow data were analyzed using FlowJo software (version 9.32, Tree Star).

### Quantitative real‑time polymerase chain reaction (qRT-PCR)

Total RNA was extracted using MolPure® Cell RNA Kit (Yeasen, Cat. no. 19231ES50) according to the manufacturer’s instructions. RNA was reverse transcribed to cDNA with TransScript® One-Step gDNA Removal and cDNA Synthesis SuperMix (TransGen, Cat. no. AT311-03). Quantitative real-time PCR was performed using AceQ qPCR SYBR Green Master Mix (Vazyme, Cat. no. Q111-02) according to the manufacturer’s protocol and run on a QuantStudio 7 Flex thermocycler (Applied Biosystems). GAPDH was used as an endogenous control. The qRT-PCR primers used in this study are listed in Supplementary Table 5.

### Tissue microarray analysis and immunohistochemistry staining

The human microarrays containing 24 glioma tissue samples and corresponding clinicopathological information were obtained from Xi’an Bioaitech.com (Cat. no. N026Ct01).

The antibody against B7-H3 (Abcam, Cat. no. ab227670) was used for immunohistochemistry (IHC) staining according to the manufacturer’s protocol. The overall immunoreactive score of each sample was defined as the product of the staining intensity and positive rate (0–300%). The staining intensity was divided into 4 stages (no signal = 0, weak signal = 1, moderate signal = 2 and strong signal = 3), and the positive rates ranged from 0 to 100%. The IHC results were analyzed using Aipathwell (Wuhan servicebio technology CO., LTD).

### RNA sequencing and data analysis

The CRISPRi-U87 cells expressing different sgRNAs were co-cultured with B7-H3 CAR T cells at an E: T ratio of 1:4 for 12 h. Following the co-culture, the CRISPRi-U87 cells were isolated and total RNA was extracted using Trizol (Invitrogen, Cat. no. 15,596,026); RNA purity and quantification were evaluated using Qubit 4.0 (Thermo Scientific); RNA integrity was assessed using Agilent 2100 Bioanalyzer (Agilent Technologies); The sequencing libraries were constructed using Hieff NGS® Ultima Dual-mode mRNA Library Prep Kit for Illumina® (Yeasen, Cat. no. 12,301). RNA-seq was performed on the DNBSEQ-T7 platform (Geneplus-Shenzhen). At least 12 Gb sequencing data (PE150) per sample were obtained.

The transcriptome sequencing data were aligned to the reference genome GRCh38 using the STAR alignment software (version 2.7.6a). Gene expression levels were quantified using the StringTie2 software (version 2.0.4). The normalization of expression levels was performed using two methods: Fragments Per Kilobase of transcript per Million mapped reads (FPKM) and Transcripts Per Million (TPM). Differential expression analysis of two conditions was performed using the DEGSeq2 R package (1.26.0). The P values were adjusted using the Benjamini & Hochberg method. A corrected P-value of 0.05 and log2(Fold change) of 1 were set as the threshold for significantly differential expression.

Gene Ontology and KEGG enrichment analysis were performed to deduce the potential biological functions by an R package-clusterProfiler (version 3.14.0). Genes with at least one read in treatment or control samples were considered the enrichment analysis background.

### Stimulation of B7-H3.CAR T cells with plate-bound recombinant B7-H3-Fc protein

Non-tissue culture-treated 24-well plates were coated with 0.5 µg/mL recombinant human B7-H3-Fc proteins with or without recombinant trimeric TL1A/TNFSF15 protein (MCE, Cat. no. HY-P78447) in 100 ng/mL or 400 ng/mL at 4 °C for 24 h. Plates were washed with DPBS and T cell medium, and 5 × 10^5^ B7-H3.CAR T cells were added onto the plate for stimulation. Cells were collected 24 h post stimulation for FACS analysis.

### Public data mining

Gene expression profiles and patient survival data of 1018 CGGA glioma samples and 751 TCGA glioma samples were obtained from the Chinese Glioma Genome Atlas (http://www.cgga.org.cn/analyse/RNA-data.jsp) and The Cancer Genome Atlas Program (https://portal.gdc.cancer.gov/). RNA sequencing data of 1079 normal brain samples were obtained from the UCSC Xena (https://xenabrowser.net/). Single-cell transcriptome data for cytokine response in mice were obtained from the Immune Dictionary (https://immune-dictionary.org/app/home).

### Statistical analysis

Statistical analysis was performed using GraphPad Prism 9 software. The Student’s t-test was used for comparison between two independent groups. One-way analysis of variance (ANOVA) was used to compare at least 3 experimental groups. The error bars represent the mean ± standard error of the mean (SEM). P values of less than 0.05 were considered statistically significant (∗< 0.05, ∗∗< 0.01 and ∗∗∗ < 0.001).

## Results

### B7-H3/CD276 is highly expressed in GBM

**Fig. 1:**
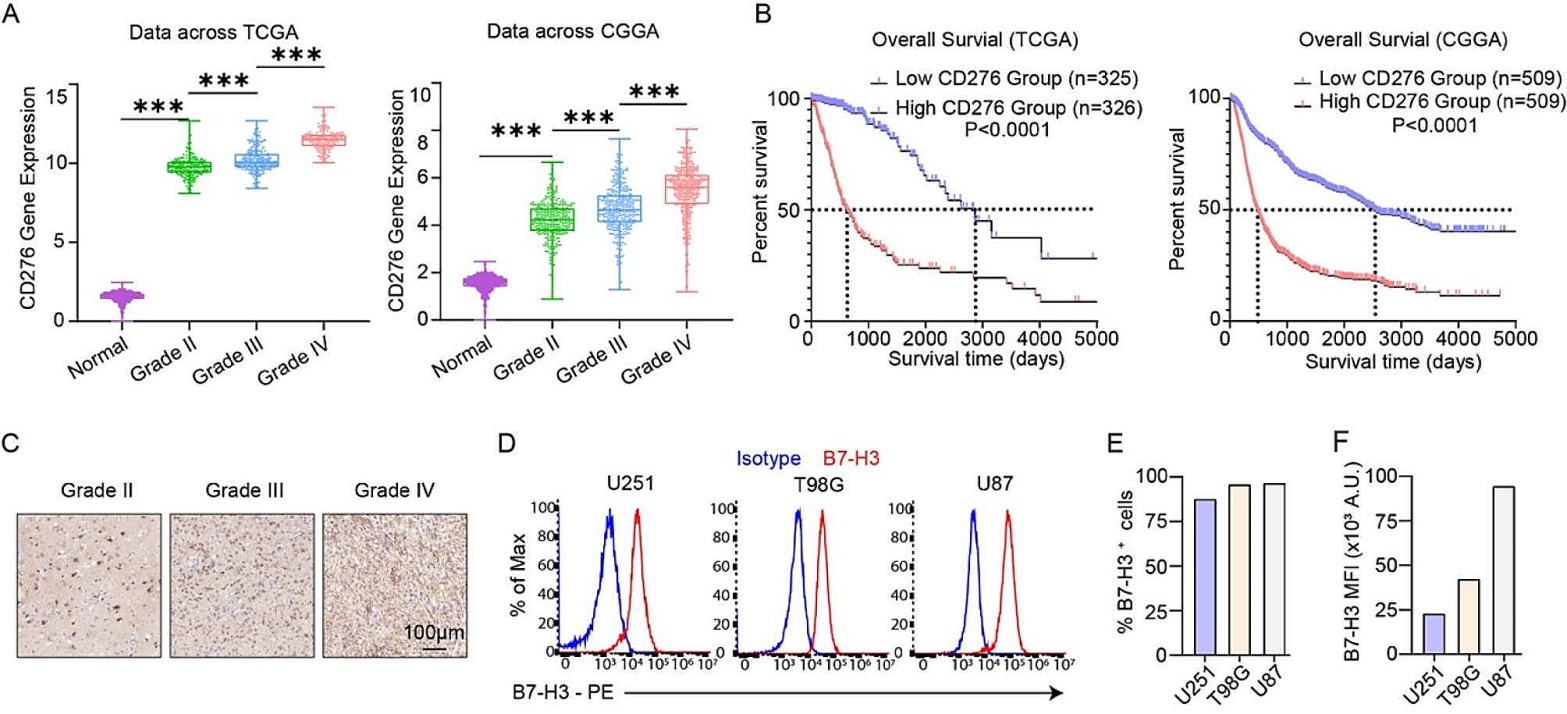
B7-H3/CD276 is highly expressed in GBM. **A** The mRNA expression levels of B7-H3/CD276 in normal brain tissues and glioma patient samples of various grades. Data were obtained from the TCGA and CGGA databases. **B** The Kaplan–Meier curves for overall survival (OS) of glioma patients with high and low expression levels of B7-H3/CD276. Data were obtained from the TCGA and CGGA databases. **C** Immunohistochemical analysis of B7-H3 expression in different grades of glioma samples. **D** Cell surface levels of B7-H3 in three human GBM cell lines stained with the B7-H3-PE antibody as measured by flow cytometry. **E&F** Quantifications of the percentage of B7-H3^+^ cells (**E**) and mean fluorescence intensity (MFI) of B7-H3 staining signal (**F**)

Our previous studies showed that B7-H3 (also known as CD276) is a tumor-associated antigen expressed on the surface of various cancer cell types and can be effectively targeted by B7-H3 specific CAR T cells [30, 50–53]. To determine whether B7-H3 can also be used as a target antigen in human GBM, we first analyzed the gene expression profiles of glioma samples in The Cancer Genome Atlas (TCGA) [54–56] and Chinese Glioma Genome Atlas (CGGA) databases [57]. We found that B7-H3 (encoded by the gene *CD276*) is highly expressed in all stages of glioma compared to normal brain tissue. Notably, its expression levels increase with increasing glioma grade, reaching the highest levels in GBM (Grade IV glioma) (Fig. 1A). Moreover, high expression levels of *CD276* are associated with significantly shortened patient survival (Fig. 1B). In addition, IHC analysis of a tissue microarray containing 24 glioma samples also confirmed the high expression of B7-H3 in various stages of glioma, including GBM (Fig. 1C, Supplementary Table 1).

Next, we determined B7-H3 expression levels in 3 commonly used GBM cell lines (U251 MG, T98G and U87 MG) by cell surface immunostaining, followed by flow cytometry analysis. Our results showed that the B7-H3 antigen is abundantly expressed in all tested cell lines, with the highest level observed in U87 MG cells (Fig. 1D-F). These data suggest that B7-H3 could be an ideal target antigen for developing CAR T therapy against GBM.

### B7-H3-specific CAR T cells recognize and eradicate GBM cells

**Fig. 2:**
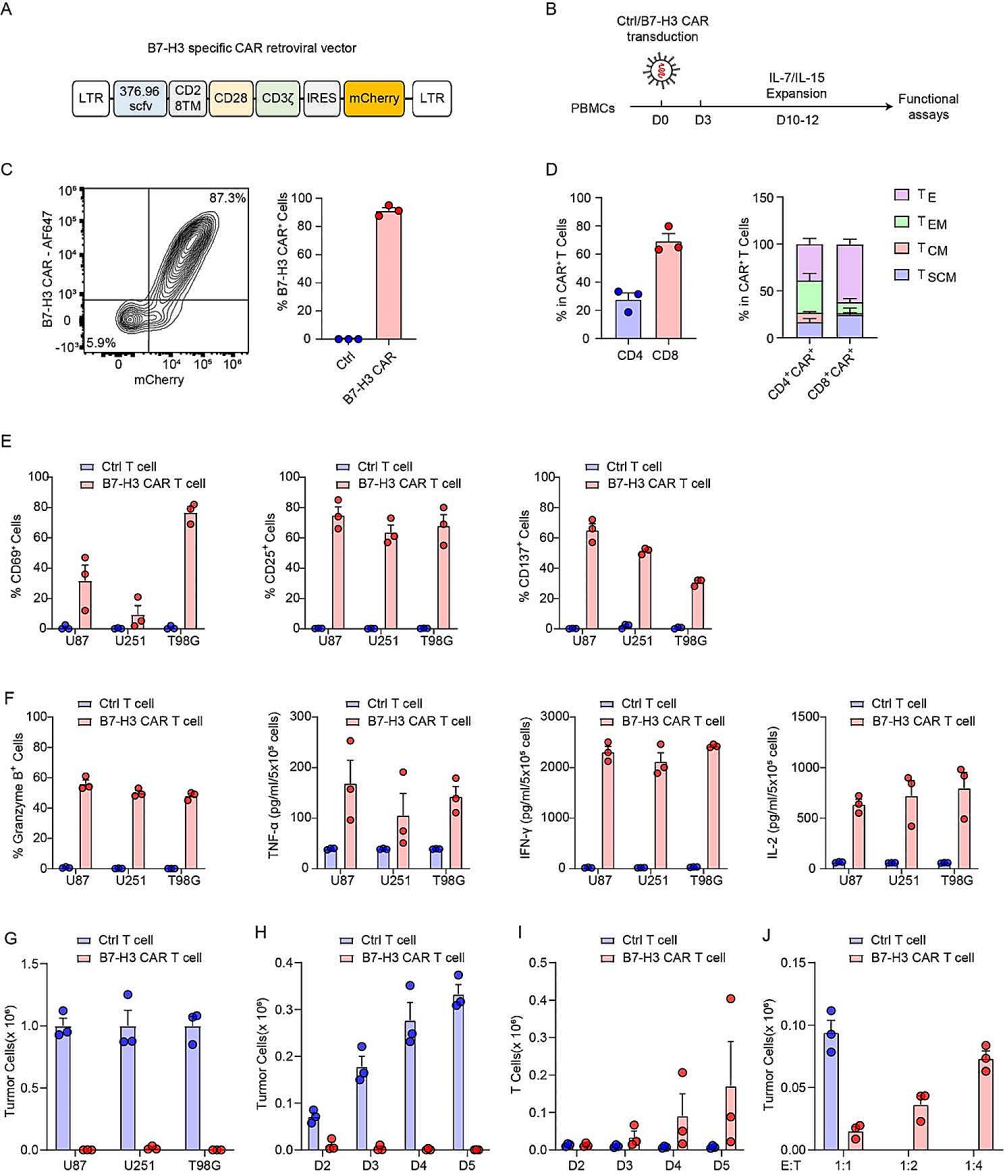
B7-H3-specific CAR T cells recognize and eradicate GBM cells. **A** Retroviral vectors construct encoding the B7-H3.CAR and bicistronic mCherry reporter. **B** Schematics of transduction and expansion of CAR T cells used in this study. **C** Expression of the B7-H3.CAR in transduced human T cells. The transduction efficiency of CAR molecules was measured either by mCherry expression or by direct staining of B7-H3 specific scFv molecules. Data shown are a representative FACS plot and quantifications of 3 independent donors. Error bars denote SEM. **D** Phenotypic analysis of CAR^+^ T cells at 12 days post transduction showing the frequency of CD4^+^ and CD8^+^ population in CAR^+^ T cells (left) as well as percentage of stem cell memory T cells (T_SCM_, CD45RA^+^CCR7^+^), central memory T cells (T_CM_, CD45RA^−^CCR7^+^), effector memory T cells (T_EM_, CD45RA^−^CCR7^−^ ), and effector T cells (T_E_, CD45RA^+^CCR7^−^ ) in CD4^+^ T cells and CD8^+^ T cells (*n* = 3). Error bars denote SEM. **E** Surface staining for CD69, CD25, and CD137 of CAR T cells after co-culture with the indicated cell lines for 24 h (*n* = 3). Error bars denote SEM. **F** Expression of granzyme B and cytokine (TNF-α, IFN-γ and IL-2) secretion of CAR T cells after co-culture with the indicated cell lines for 24 h (*n* = 3). Error bars denote SEM. **G** Counts of residual tumor cell lines after 5-day co-culture with CAR T cells or Ctrl T cells at 1:1 E: T ratio (*n* = 3). Error bars denote SEM. **H&I** Counts of U87 MG tumor cells and T cells (CAR T or Ctrl T cells) on indicated days post co-culture at 1:1 E: T ratio (*n* = 3). Error bars denote SEM. **J** Counts of U87 MG tumor cells on day two post co-culture with CAR T cells or Ctrl T cells at 1:1, 1:2, and 1:4 E: T ratio (*n* = 3). Error bars denote SEM

We generated B7-H3-specific CAR T cells by transducing primary human T cells with a bicistronic retroviral vector encoding CAR molecule with CD28 and CD3ζ endo-domains and mCherry as a detection marker (Fig. 2A**&B**). The CAR construct was efficiently expressed, as determined by mCherry expression and direct staining of CAR proteins on the cell surface (Fig. 2C). Ex vivo cultured B7-H3-specific CAR T cells contained 27.8% and 69.3% CD4^+^ and CD8^+^ cells, respectively and consist of memory T cell populations consistent with previous studies (Fig. 2D, Supplementary Fig. 1) [51]. Our B7-H3-specific CAR T cells exhibit strong T cell activation, indicated by the expression of activation markers CD69, CD25 and CD137 when co-cultured with GBM cell lines (Fig. 2E). Furthermore, expression of the cytotoxic protein granzyme B and cytokine secretion were robustly and specifically induced upon tumor engagement (Fig. 2F).

To assess the cytotoxic efficacy of B7-H3-specific CAR T cells against GBM cells, we co-cultured three GBM cell lines with either CAR T cells or untransduced T cells (Ctrl) at 1-to-1 effector-to-target (E: T) ratio for 5 days. The residual tumor cells were quantified using flow cytometry. Remarkably, all three cell lines were efficiently eradicated by CAR T cells but not by Ctrl T cells (Fig. 2G). Next, we determined the kinetics of CAR T cell-mediated killing of U87 MG cells and found that tumor eradication was apparent starting from day 2 of co-culture (Fig. 2H), while CAR T cells gradually expanded over time (Fig. 2I). Furthermore, we observed increased tumor killing as a function of E: T ratios, with tumor cell elimination increasing from approximately 20% to approximately 90% as the E: T ratio increased from 1:4 to 1:1 (Fig. 2J). These data demonstrate that GBM cell lines can be effectively targeted by B7-H3-specific CAR T cells.

### A co-culture CRISPRi screen identified regulators of B7-H3 CAR T cell-mediated cytotoxicity in GBM cells

**Fig. 3:**
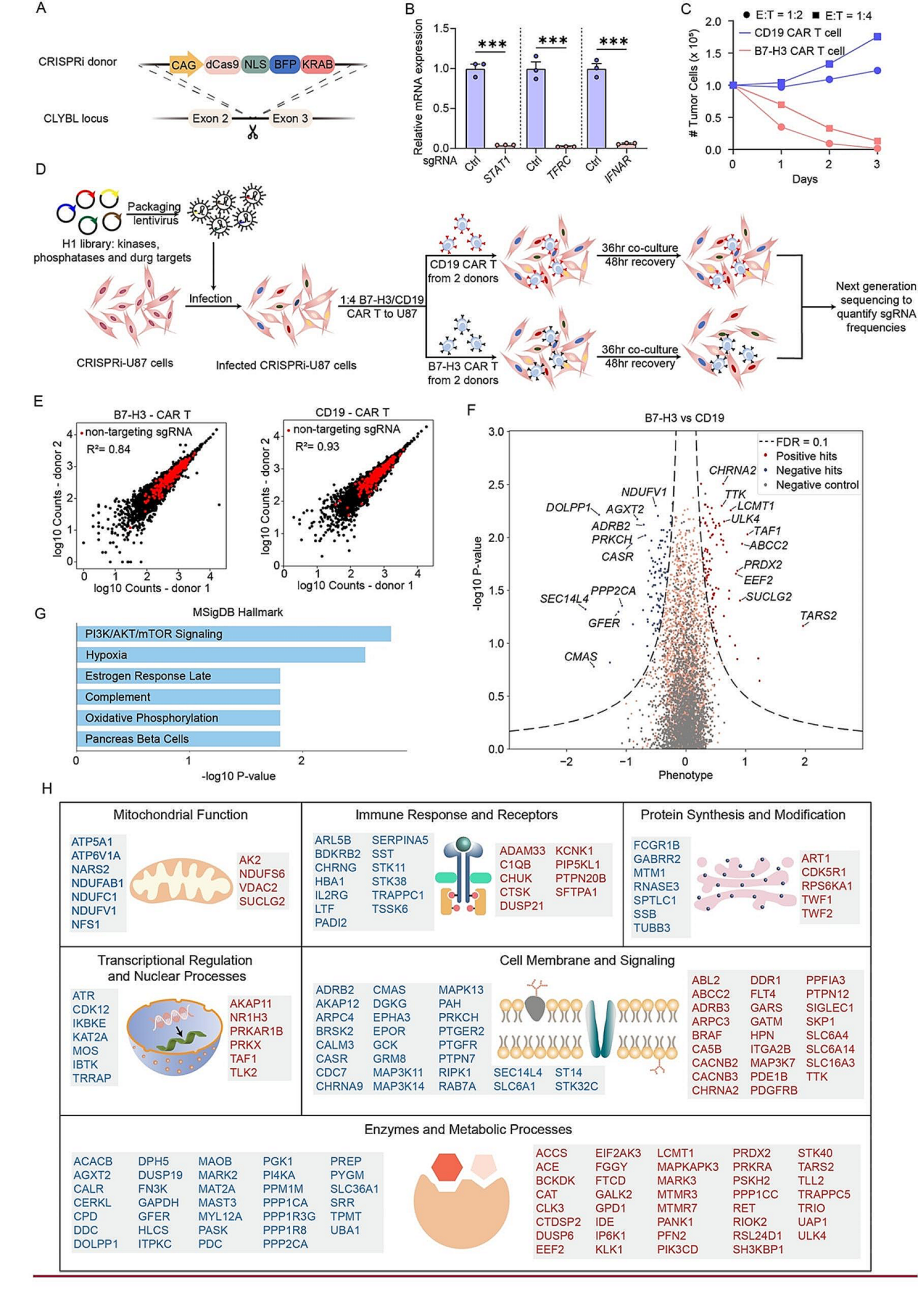
A co-culture CRISPRi screen identified regulators of B7-H3 CAR T cell-mediated cytotoxicity in GBM cells. **A** The construct for expressing the CRISPRi machinery from the CLYBL safe-harbor locus: catalytically dead Cas9 (dCas9) fused to a blue fluorescent protein (BFP) and the KRAB domain, under the control of the constitutive CAG promoter. **B** Functional validation of CRISPRi activity in the CRISPRi-U87 cells via qPCR with sgRNAs targeting *STAT1*, *TFRC* and *IFNAR*. A non-targeting sgRNA was used as the control. **C** Killing effect of B7-H3 and CD19 CAR T cells against U87 MG cells at an E: T ratio of 1:2 or 1:4 after 1-, 2- or 3-day co-culture. **D** Schematic of the co-culture CRISPRi screen. CRISPRi-U87 cells were transduced with the H1 sgRNA library and co-cultured with CD19 or B7-H3 targeting CAR T cells at an E: T ratio of 1:4 for 36 h, followed by a 48-hour recovery. Frequencies of CRISPRi-U87 cells expressing a given sgRNA were determined in each population by next-generation sequencing. The screens were performed in parallel with CAR T cells derived from 2 donors as biological replicates. **E** Correlations of sgRNA counts between two donors of B7-H3 (left) and CD19 (right) CAR T screening groups. **F** Volcano plots summarizing phenotypes and statistical significances of gene perturbations in the screen as determined by the MAGeCK-iNC pipeline. Dashed line indicates the cut-off for hit genes (false discovery rate (FDR) = 0.1). Dots in blue or red represent hit genes whose knockdown enhanced or suppressed CAR T cytotoxicity, respectively. Non-hit genes are shown in orange and negative controls are shown in gray. Gene symbols of the top ten negative hits and the top ten positive hits are shown. **G** Enrichment analysis of the negative screening hits against gene sets in the Human Molecular Signatures Database (MSigDB). Enriched terms with adjusted P values less than 0.05 are shown. **H** Screening hits grouped by their biological function. Gene symbols in blue and red represent genes whose knockdown in U87 MG cells represses and enhances CAR T cytotoxicity, respectively

Next, we sought to identify genetic modifiers in GBM cells that modulate their susceptibility to B7-H3 CAR T cell-mediated killing. To this end, we engineered a U87 MG cell line that stably expresses the CRISPRi machinery (hereafter referred to as CRISPRi-U87) by integrating a CAG promoter-driven dCas9-BFP-KRAB expression cassette into the CLYBL safe harbor locus through homologous recombination [46] **(**Fig. 3A**)**. Robust gene silencing was observed in this cell line when testing with three previously validated sgRNAs targeting *STAT1*, *TFRC* and *IFNAR*, respectively [46, 48] (Fig. 3B).

We transduced CRISPRi-U87 cells with the H1 library, which comprises approximately 13,000 sgRNAs targeting 2,318 genes encoding kinases, phosphatases, and drug targets with 5 or 10 sgRNAs per gene, along with 500 non-targeting control sgRNAs [49]. Subsequently, the cells were co-cultured with either B7-H3 or CD19 CAR T cells. CD19 CAR T cells were used as a control because CD19 is not expressed in GBM cells, and CD19 CAR T cells did not exhibit cytotoxicity towards U87 MG cells (Fig. 3C). We determined that a co-culture duration of 36 h and a E: T ratio of 1:4 resulted in approximately 50% tumor cell killing, which would allow us to identify genetic modifiers that either enhance or suppress the susceptibility of U87 MG cells to CAR T cell cytotoxicity (Fig. 3C). After co-culture, CAR T cells were removed and CRISPRi-U87 cells were recovered for 48 h. The remaining cells were then harvested and processed for next-generation sequencing to determine sgRNA abundancies (Fig. 3D). The screens were performed in parallel with CAR T cells derived from two separate donors. sgRNA abundances were compared between the B7-H3 and CD19 groups to determine the phenotype and significance of each gene perturbation using the MAGeCK-iNC pipeline [58].

The screen results demonstrated high reproducibility, as evidenced by the strong correlation between the two donor replicates (Fig. 3E). We identified numerous positive and negative hits, corresponding to genes whose knockdown suppressed or enhanced the susceptibility of U87 MG cells to CAR T cell cytotoxicity, respectively (Fig. 3F, Supplementary Tables 2 and 3). These hits covered diverse cellular processes, and the negative hits were enriched in pathways including the PI3K/AKT/mTOR signaling, Hypoxia and Oxidative phosphorylation (Fig. 3G&H).

### Knockdown of high-confident screening hits in GBM enhanced B7-H3 CAR T cell-mediated killing by stimulating cytotoxic Granzyme B production

**Fig. 4:**
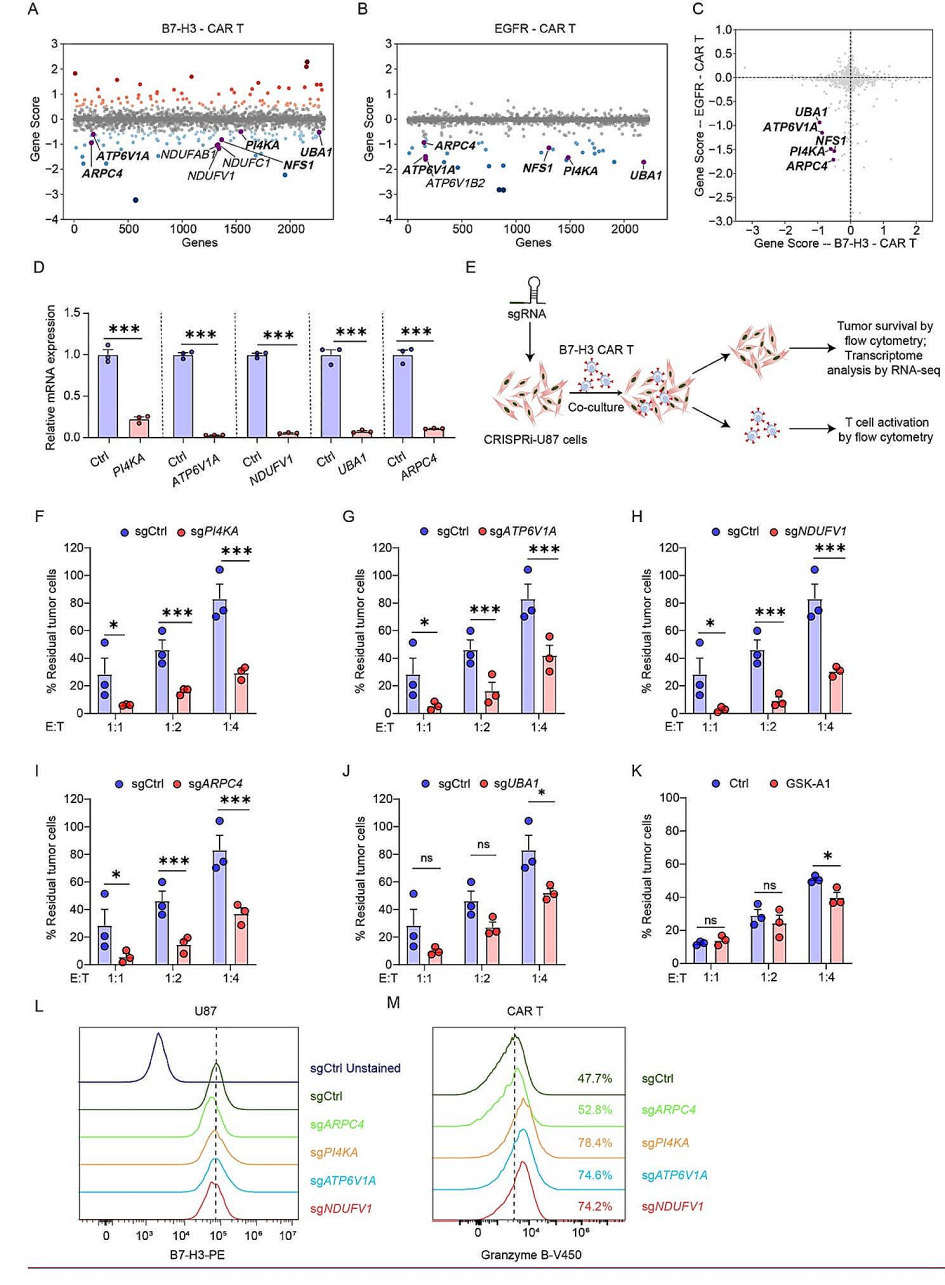
Identification of high-confident hits and validation of their knockdown effect in enhancing CAR T cell-mediated tumor killing. **A-C** Comparing screening results in U87 MG cells that were co-cultured with B7-H3-targeting CAR T cells (**A**, this study) and with EGFR-targeting CAR T cells (**B**, Larson et al. [41]) revealed common hits showing CAR T enhancing phenotypes (**C**). **D** Knockdown of *PI4KA*, *ATP6V1A*, *NDUFV1*, *UBA1* and *ARPC4* in CRISPRi-U87 cells. The relative mRNA level of each targeted gene was calculated as the ratio of its expression in cells expressing a targeting sgRNA as compared to a non-targeting control sgRNA measured by qPCR. Error bars denote SEM. **E** Strategies for further characterization of the selected high-confident hits. **F-J** Tumor killing effect of B7-H3 CAR T cells against CRISPRi-U87 cells with *PI4KA* (**F**), *ATP6V1A* (**G**), *NDUFV1* (**H**), *ARPC4* (**I**) and *UBA1* (**J**) knockdown after 2-day co-culture at E: T ratios of 1:1,1:2, and 1:4 (*n* = 3). Error bars denote SEM. **K** Tumor killing effect of B7-H3 CAR T cells against U87 MG cells treated with vehicle (Ctrl) or 5 nM PI4KA inhibitor GSK-A1 at E: T ratios of 1:1, 1:2 and 1:4. Data shown are the percentages of residual tumor cells after 2-day co-culture (*n* = 3). Error bars denote SEM. **L** Cell surface levels of B7-H3 in CRISPRi-U87 cells expressing the indicated sgRNAs stained with the B7-H3-PE antibody as assessed by flow cytometry. **M** Intracellular staining of granzyme B in CAR T cells after 24 h co-culture with the CRISPRi-U87 cells expressing the indicated sgRNAs as assessed by flow cytometry

Our screens identified multiple genes that encode components of mitochondrial complex I, including *NDUFAB1*, *NDUFC1* and *NDUFV1*. Knockdown of these genes enhanced the susceptibility of U87 MG cells to B7-H3 CAR T cell killing, suggesting an important role of complex I in this process (Fig. 4A, Supplementary Tables 2&3). Furthermore, we compared our screening results with a previously published CRISPR screen in U87 MG cells focused on EGFR CAR T cell-mediated killing [41]. We re-analyzed the EGFR CAR T screening data with the MAGeCK-iNC pipeline and filtered out genes not in the H1 library (Fig. 4B, Supplementary Table 4). The comparison revealed five overlapping negative hit genes: *ARPC4*, *ATP6V1A*, *NFS1*, *PI4KA* and *UBA1* (Fig. 4A-C, Supplementary Fig. 2). Based on these analyses, we prioritized five genes, *NDUFV1*, *ARPC4*, *ATP6V1A*, *PI4KA*, and *UBA1*, as high-confidence hits for further validation.

We generated knockdown cell lines in CRISPRi-U87 cells by individually cloning sgRNAs targeting these genes (Fig. 4D). Subsequently, these cell lines were co-cultured with CAR T cells to assess cell survival and analyze gene expression of tumor cells, as well as evaluate T cell activation of CAR T cells (Fig. 4E). Remarkably, knockdown of *PI4KA*, *ATP6V1A*, *NDUFV1* and *ARPC4* significantly enhanced B7-H3 CAR T cell killing at all E: T ratios tested, as compared to control sgRNA (sgCtrl) (Fig. 4F-I). Knockdown of *UBA1* also showed a significant effect at an E: T ratio of 1:4 (Fig. 4J). Notably, the number of CAR T cells at the end of co-culture was unchanged (Supplementary Fig. 3), suggesting the increased sensitivity was not due to greater proliferation or expansion of CAR T cells but more likely to elevated per-cell killing capacity. Importantly, a selective PI4KA inhibitor, GSK-A1 [59], also increased CAR T cytotoxicity at a low E: T ratio (Fig. 4K). Collectively, these data validate the effect of high-confidence screening hits in enhancing CAR T cell efficacy against GBM, highlighting the robustness of our CRISPRi screen.

Next, we aimed to elucidate the mechanisms underlying the increased CAR T cell susceptibility after knocking down the high-confident hit genes in GBM cells. We excluded *UBA1* from further characterization due to its high toxicity upon knockdown in U87 MG cells. Immunostaining analysis showed no increase in cell surface B7-H3 levels in any of the knockdown cell lines compared to the control (Fig. 4L), indicating that increased CAR T killing was not due to elevated antigen expression. Next, we determined the production of cytotoxic molecule granzyme B and the expression of activation markers in CAR T cells upon their engagement with different knockdown cell lines. Interestingly, Granzyme B production was elevated in all knockdown cell lines, with the most dramatic increase observed in *PI4KA, ATP6V1A* and *NDUFV1* knockdown cells (Fig. 4M). These data suggest that knockdown of the selected high-confident hit genes potentiates CAR T cell cytotoxicity through stimulating Granzyme B production in CAR T cells.

### Upregulated cytokine signaling as a convergent mechanism mediating the effect of *ARPC4* and *NDUFV1* knockdown in enhancing CAR T cell cytotoxicity

**Fig. 5:**
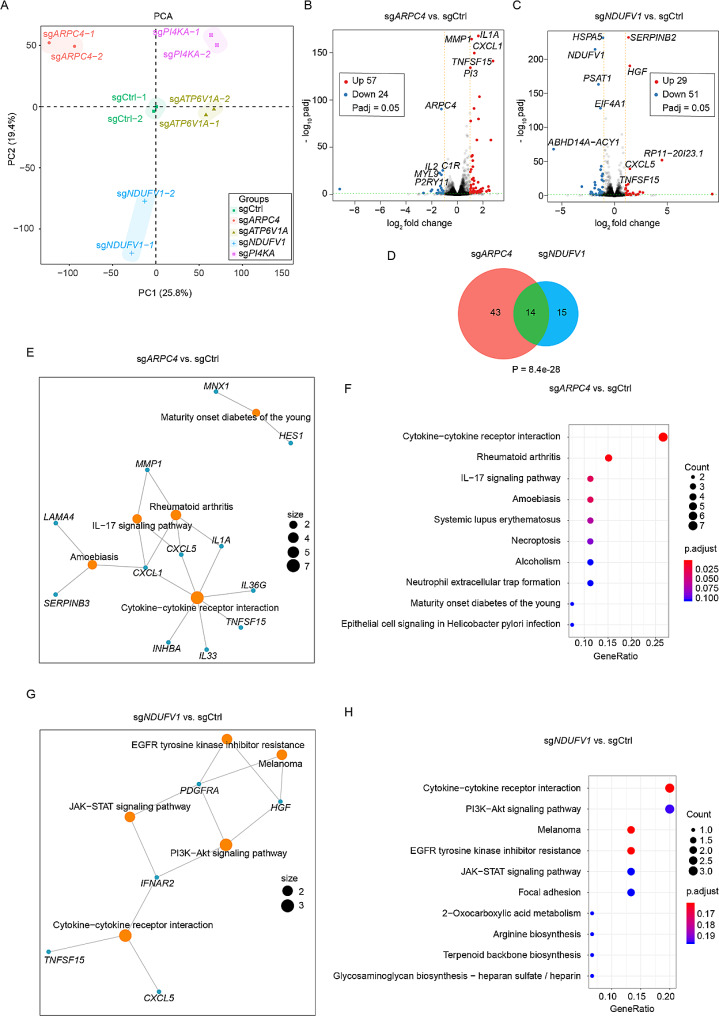
Transcriptome analysis revealed common gene expression signatures in U87 MG cells upon knocking down ARPC4 and NDUFV1. **A** Principal Component Analysis (PCA) on the expression profiles of CRISPRi-U87 cells expressing indicated sgRNA after 12-hour co-culture with B7-H3 CAR T cells. Each data point corresponds to an independent biological replicate, with colors denoting distinct gene knockdown groups. **B&C** Volcano plots showing differentially expressed genes following the knockdown of *ARPC4* (**B**) and *NDUFV1* (**C**) in CRISPRi-U87 cells. **D** Venn diagram illustrating the overlap of upregulated genes between the knockdown of *ARPC4* and *NDUFV1* in CRISPRi-U87 cells. **E&F** KEGG pathway enrichment analysis on upregulated genes in *ARPC4* knockdown cells. **G&H** KEGG pathway enrichment analysis on upregulated genes in *NDUFV1* knockdown cells

To understand how the knockdown of hit genes in U87 MG cells activates granzyme B production in CAR T cells, we performed RNA-seq analyses on CRISPRi-U87 cells expressing either a control sgRNA or sgRNAs targeting *ARPC4*, *ATP6V1A*, *NDUFV1* and *PI4KA* after co-cultured with B7-H3 CAR T cells for 12 h at an E: T ratio of 1:1 (Fig. 4E). The analyses revealed that cells with different gene knockdown exhibited distinct gene expression profiles (Fig. 5A-C, Supplementary Fig. 4). Intriguingly, the knockdown of *ARPC4* and *NDUFV1*, two genes with distinct known functions, resulted in a substantial overlap of upregulated genes (Fig. 5B-D). In particular, the upregulated genes were enriched in the KEGG pathway “Cytokine-cytokine receptor interaction” in both knockdown groups (Fig. 5E-H). This finding suggests that the knockdown of *ARPC4* and *NDUFV1* may have a convergent impact on cytokine signaling pathways, potentially contributing to the observed activation of Granzyme B production in CAR T cells.

### TNFSF15-mediated activation of the NF-κB pathway enhances CAR T cell cytotoxicity

**Fig. 6:**
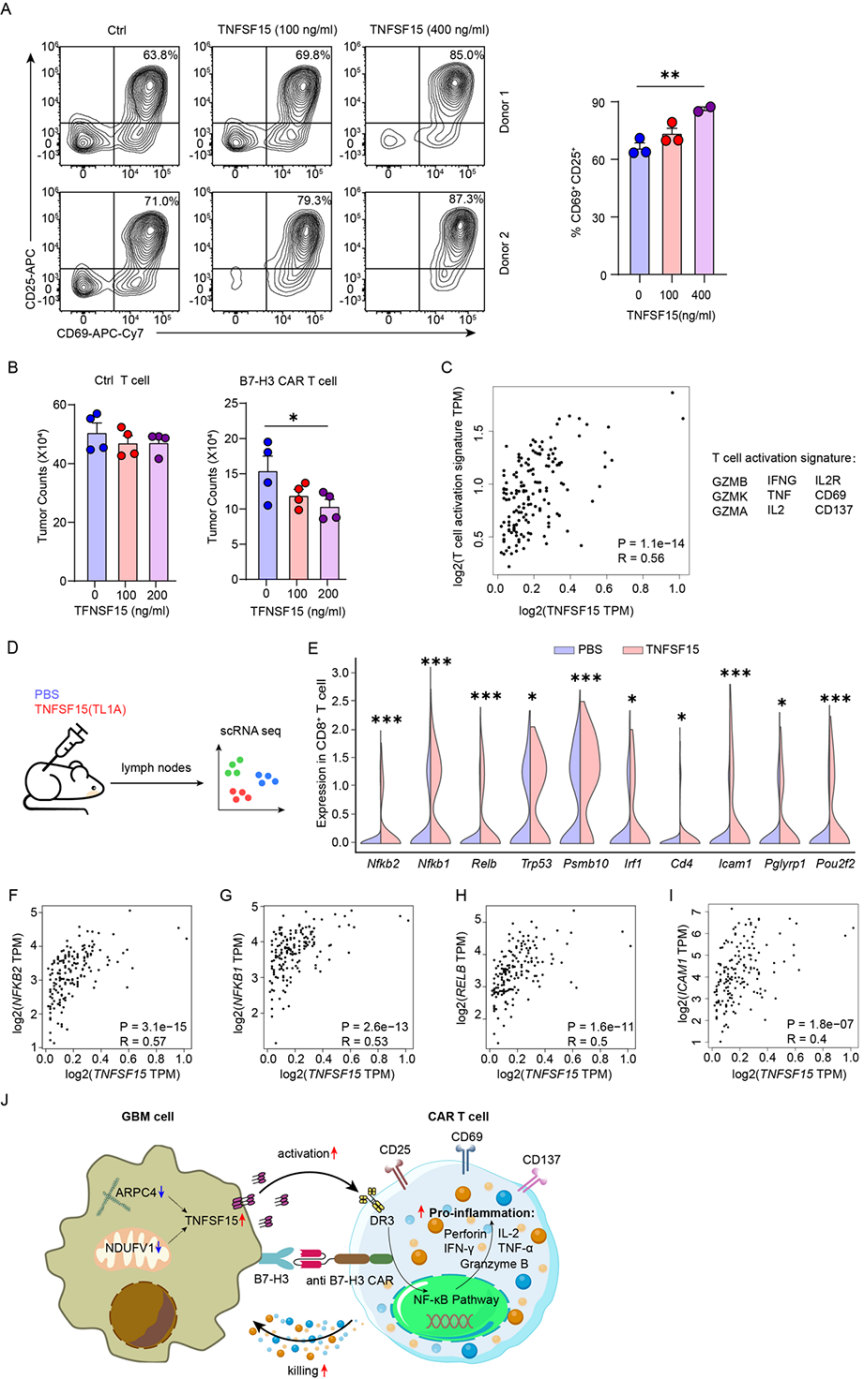
TNFSF15 is an immunostimulatory factor that enhances CAR T cell cytotoxicity. **A** Cell surface staining of CD69 and CD25 in CAR T cells stimulated by plate-bound recombinant B7-H3-Fc protein with or without recombinant trimeric TNFSF15 protein. (*n* = 3 for 0, 100 ng/mL and = 2 for 400 ng/mL). Error bars denote SEM. **B** Tumor killing of U87 MG cells by B7-H3-targeting CAR T cells or control T cells at an E: T ratio of 1:2 after two-day co-culture with or without exogenous addition of recombinant TNFSF15 protein (*n* = 4). Error bars denote SEM. **C** Gene expression correlation between *TNFSF15* and the T cell activation signature (*GZMB*、*GZMK*、*GZMA*、*IFNG*、*TNF*、*IL2*、*IL2R*、*CD69* and *CD137*) in human GBM samples. Data were obtained from TCGA. **D** Schematic of a recent study that performed single-cell RNA sequencing on mouse skin-draining lymph nodes for probing cellular response to various cytokines, including TNFSF15 (TL1A). **E** Violin plots showing the expression levels of genes involved in T cell activation and NF-κB pathway in CD8^+^ T cells following PBS or TNFSF15 treatment in vivo. **F-I** Gene expression correlation between *TNFSF15* and *NFKB2* (**F**), *NFKB1* (**G**), *RELB* (**H**) and *ICAM1* (**I**) in human GBM samples. Data were obtained from TCGA. **J** Schematic of our proposed model. Inhibiting *ARPC4* or *NDUFV1* in GBM cells upregulates TNFSF15. TNFSF15 acts as an immunostimulatory factor that activates the NF-κB pathway in CAR T cells, leading to increased production and release of proinflammatory and cytotoxic factors, thus enhancing the anti-tumor activity of CAR T cells

Our RNA-seq analyses revealed *TNFSF15* as one of the shared upregulated genes involved in cytokine signaling in both *APRC4* and *NDUFV1* knockdown cells **(**Fig. 5B-H**)**. Previous studies showed that TNFSF15 engages with the DR3 receptor (encoded by the *TNFRSF25* gene) on T cells and activates NF-κB and MAPK signaling cascade through TRAF2 [60]. In addition, TNFSF15 upregulation was observed in various autoimmune diseases, such as rheumatoid arthritis and inflammatory bowel disease [61]. Thus, the upregulated TNFSF15 in tumor cells upon *APRC4* and *NDUFV1* knockdown may serve as an immune-stimulatory factor for CAR T cells, facilitating their tumor lytic activity. To test this hypothesis, we first activated B7-H3 CAR T cells with a plate-bound recombinant B7-H3-Fc protein with or without recombinant trimeric TNFSF15 protein, and measured T cell activation 24 h post activation. Intriguingly, the addition of TNFSF15 protein exhibited a significant and dose-dependent increase of T cell activation based on the expression of CD69 and CD25 (Fig. 6A). Moreover, the addition of recombinant trimeric TNFSF15 protein into CAR T-tumor coculture significantly promoted tumor killing by CAR T cells in an antigen-specific and dose-dependent manner (Fig. 6B). To corroborate with these observations, we leveraged public transcriptomics data of human GBM samples in TCGA and found a strong positive correlation between *TNFSF15* expression and T cell activation signature (Fig. 6C). Taken together, these data support our hypothesis that upregulated TNFSF15 in *APRC4* and *NDUFV1* knockdown GBM cells plays an immune-stimulatory role in enhancing CAR T cell efficacy.

To further elucidate the mechanisms by which TNFSF15 stimulates CAR T cells, we performed single-cell transcriptome analysis on a recently published comprehensive database of in vivo cellular immune responses to cytokines [62]. In that study, mice were treated with either PBS control or different cytokines, followed by single-cell RNA sequencing of their skin-draining lymph nodes [62]. We specifically examined the gene expression changes within the CD8^+^ T cells upon TNFSF15 (also known as TL1A) injection compared to PBS (Fig. 6D). Interestingly, we discovered that TNFSF15 treatment induced the upregulation of many genes involved in immune function, with the top 50 upregulated genes enriched in GO terms “response to cytokine”, “regulation of immune system process” and “lymphocyte activation” (Supplementary Fig. 5). In particular, genes in the NF-κB pathway, including *Nfkb1*, *Nfkb2* and *Relb*, and genes associated with T cell activation, including *Trp53*, *Psmb10*, *Irf1*, *Cd4*, *Icam1*, *Pglyrp1*, and *Pou2f2* were significantly upregulated (Fig. 6E). Supporting this finding, analysis of the TCGA database revealed strong positive correlations between the expression of *TNFSF15* and NF-κB pathway-related genes *NFKB1*, *NFKB2* and *RELB*, as well as a key marker gene associated with T cell activation *ICAM1* in human GBM samples [63] (Fig. 6F-I).

In summary, our study proposes the following model: knockdown of *APRC4* or *NDUFV1* in GBM cells leads to the upregulation of the immuno-stimulatory factor TNFSF15 in tumor cells. This, in turn, promotes the activation of CAR T cells, stimulating the production of proinflammatory and cytotoxic factors, including granzyme B, perforin, IL-2, TNF and IFN-γ possibly through the NF-κB pathway, thereby augmenting the killing efficacy of CAR T cells against GBM (Fig. 6J).

## Discussion

CAR T cell therapy has emerged as a groundbreaking approach for cancer treatment. However, despite its remarkable efficacy in certain hematologic malignancies such as acute lymphoblastic leukemia (ALL) and diffuse large B-cell lymphoma (DLBCL), the application of CAR T cell therapy in solid tumors has faced significant challenges [64, 65]. One key challenge is the lack of tumor-specific antigens that can be effectively targeted by CAR T cells, as solid tumors often exhibit antigen heterogeneity. Additionally, the immune-resistance mechanism in solid tumors can hamper the function and persistence of CAR T cells, limiting their effectiveness [66].

In this study, we aimed to identify novel strategies to enhance CAR T cell therapy efficacy in treating GBM, a highly malignant brain tumor that exhibits limited response to conventional chemotherapy and radiotherapy. We first established B7-H3 as a targetable antigen for CAR T therapy against GBM based on (1) its tumor-specific high expression in GBM cell lines and patient samples (Fig. 1), and (2) its ability in engaging and activating B7-H3 targeting CAR T cells for tumor clearance (Fig. 2). These results are in line with previously published studies [30, 67].

Next, we employed large-scale CRISPRi screening to identify targets in GBM cells that could enhance their susceptibility to B7-H3 CAR T cell-mediated killing. Similar screens on GBM sensitivity to CAR T cells were previously conducted by Wang et al. [42] and Larson et al. [41], using GBM stem cells (GSCs) co-cultured with IL13Rα2 CAR T cells and U87 MG cells co-cultured with EGFR CAR T cells, respectively. We reasoned that common hits identified from different screens would be more reliable modifiers of CAR T therapy. Thus, we compared our results with those of Larson et al. since both studies were conducted in U87 MG cells. The comparison led to the identification of *ARPC4*, *PI4KA*, *ATP6V1A, and UBA1*, whose knockdown could improve the killing of U87 MG cells by both EGFR and B7-H3 CAR T cells. We also prioritized mitochondrial complex I subunit *NDUFV1* for further characterization as multiple mitochondrial complex I subunit genes were identified in our screen.

All five selected hits were validated to enhance CAR T cell-mediated killing upon knockdown in U87 MG cells by subsequent characterization. Their knockdown led to more cytotoxic granzyme B production in CAR T cells without changing the cell surface B7-H3 antigen levels in U87 MG cells.

One intriguing finding in our study is that genes with seemingly no related function could influence CAR T cell efficacy through a convergent mechanism. Specifically, we found that knockdown of *ARPC4*, which encodes a subunit of the Arp2/3 complex mediating actin polymerization, and *NDUFV1*, which encodes a subunit of mitochondrial complex I participating in oxidative phosphorylation, led to the upregulation of highly overlapping sets of genes. This finding suggests the presence of a shared regulatory pathway or signaling network that connects these seemingly disparate cellular processes, which requires further investigation.

Among the genes upregulated in both *APRC4* and *NDUFV1* knockdown cells, we focused on *TNFSF15*, which has been characterized as a T cell co-stimulator [68]. By integrating experimental data with bioinformatic analyses of published single-cell transcriptome and TCGA databases, we demonstrated that TNFSF15 acts as an immunostimulatory factor that promotes the production of proinflammatory and cytotoxic factors in CAR T cells, possibly through the NF-κB pathway, leading to enhanced tumor killing (Fig. 6J). Our study provides novel insights into improving CAR T cell tumor killing by modulating the tumor-CAR T interaction through intervening specific targets in cancer cells.

As our screen utilized the H1 sgRNA library that enriched for drug target genes, some of the identified hits can be directly targeted using readily available small molecules, facilitating their clinical translation as new therapeutic targets [48]. For instance, we demonstrated the potential of GSK-A1, a selective PI4KA inhibitor, in enhancing the efficacy of CAR T cell-mediated killing of GBM cells (Fig. 4K**)**. Further studies using sgRNA libraries targeting other domains of cellular processes or using genome-wide sgRNA libraries will uncover additional regulators of GBM susceptibility to CAR T cell-mediated killing.

Tumors can develop resistance to CAR T cell therapy through various mechanisms, such as genetic loss of antigen, trogocytosis or loss of death receptor signaling [64]. While our study primarily focused on factors influencing the initial efficacy of CAR T cell-mediated killing, further research is warranted to determine if the hits identified are implicated in the resistance to CAR T cell therapy. Moreover, conducting new screens specifically designed to identify genes and pathways involved in the resistance phenotype could provide valuable insights into potential strategies for overcoming or preventing the emergence of resistance to CAR T cell therapy in GBM patients.

## Conclusion

Our study demonstrated B7-H3 as a viable antigen for CAR T therapy in GBM. We identified five genes (*ARPC4*, *PI4KA*, *ATP6V1A*, *UBA1*, and *NDUFV1*) whose knockdown in GBM improved CAR T cell killing. We discovered that TNFSF15 is upregulated in both *ARPC4* and *NDUFV1* knockdown cells and acts as an immunostimulatory factor that enhances CAR T cell efficacy. These findings provide new insights into the mechanisms underlying CAR T cell-mediated tumor killing and identify potential targets for improving CAR T cell therapy in GBM and other solid tumors. Our study highlights the power of CRISPR-based genetic screening in investigating tumor-CAR T interactions and contributes to the development of novel strategies to enhance CAR T cell therapy in solid tumors.

### Electronic supplementary material

Below is the link to the electronic supplementary material.

Supplementary Material 1

Supplementary Material 2

Supplementary Material 3

Supplementary Material 4

Supplementary Material 5

Supplementary Material 6

## Data Availability

All data generated or analyzed during this study are included in this article.

## Abbreviations

GBM: Glioblastoma multiforme
CRISPRi: CRISPR interference
CAR T: Chimeric Antigen Receptor T
CRISPRn: CRISPR knockout
CRISPRa: CRISPR activation
PEI: Polyethylenimine Linear
PBMC: peripheral blood mononuclear cells
BFP: blue fluorescent protein
FACS: fluorescence-activated cell sorting
MOI: multiplicity of infection
qRT-PCR: Quantitative Real-Time Polymerase Chain Reaction
IHC: immunohistochemistry
FPKM: Fragments Per Kilobase of transcript per Million mapped reads
TPM: Transcripts Per Million
RNA-seq: RNA sequencing
ANOVA: One-way analysis of variance
SEM: standard error of the mean
TCGA: The Cancer Genome Atlas
CGGA: Chinese Glioma Genome Atlas
ALL: acute lymphoblastic leukemia
DLBCL: diffuse large B-cell lymphoma
GSC: GBM stem cell
