# Supplementary material for: Identification of genetic modifiers enhancing B7-H3-targeting CAR T cell therapy against glioblastoma through large-scale CRISPRi screening
Source: J Exp Clin Cancer Res. 2024 Apr 1;43:95. doi: 10.1186/s13046-024-03027-6 (PMC10986136; doi:10.1186/s13046-024-03027-6)
Supplement: Supplementary file 6 — Supplementary Material 6 [file 13046_2024_3027_MOESM6_ESM.docx]

**Supplementary Figures**


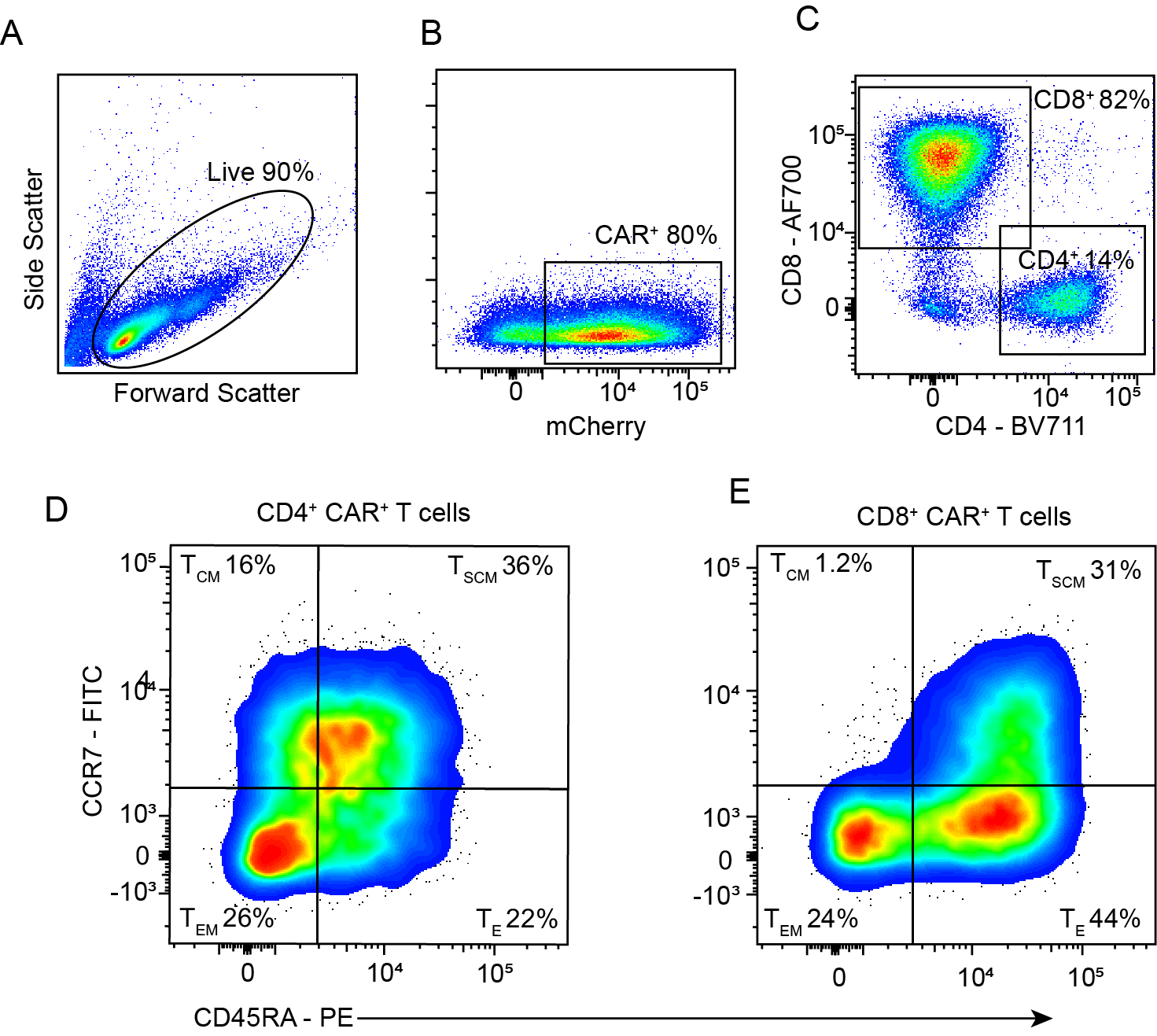


**Supplementary Figure 1. Subpopulations within B7-H3 CAR T cells**

Flow cytometry diagrams of CAR^+^ T cells at 12 days post transduction showing the frequency of CD4^+^ and CD8^+^ population in CAR^+^ T cells as well as the percentage of stem cell memory T cells (T_SCM_, CD45RA^+^CCR7^+^), central memory T cells (T_CM_, CD45RA^-^CCR7^+^), effector memory T cells (T_EM_, CD45RA^-^CCR7^-^ ), and effector T cells (T_E_, CD45RA^+^CCR7^-^ ) in CD4+ T cells and CD8+ T cells (n=3).


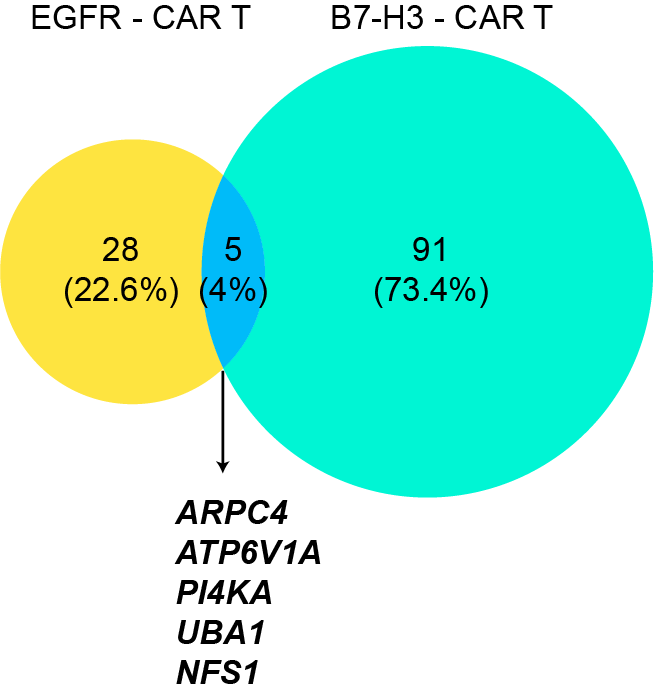


**Supplementary Figure 2. Comparison of results from our screen and a previous published screen**

Venn diagram showing the overlap between the sensitizing hits identified from our screen using B7-H3-targeting CAR T cells and the screen by Larson et al. using EGFR-targeting CAR T cells.


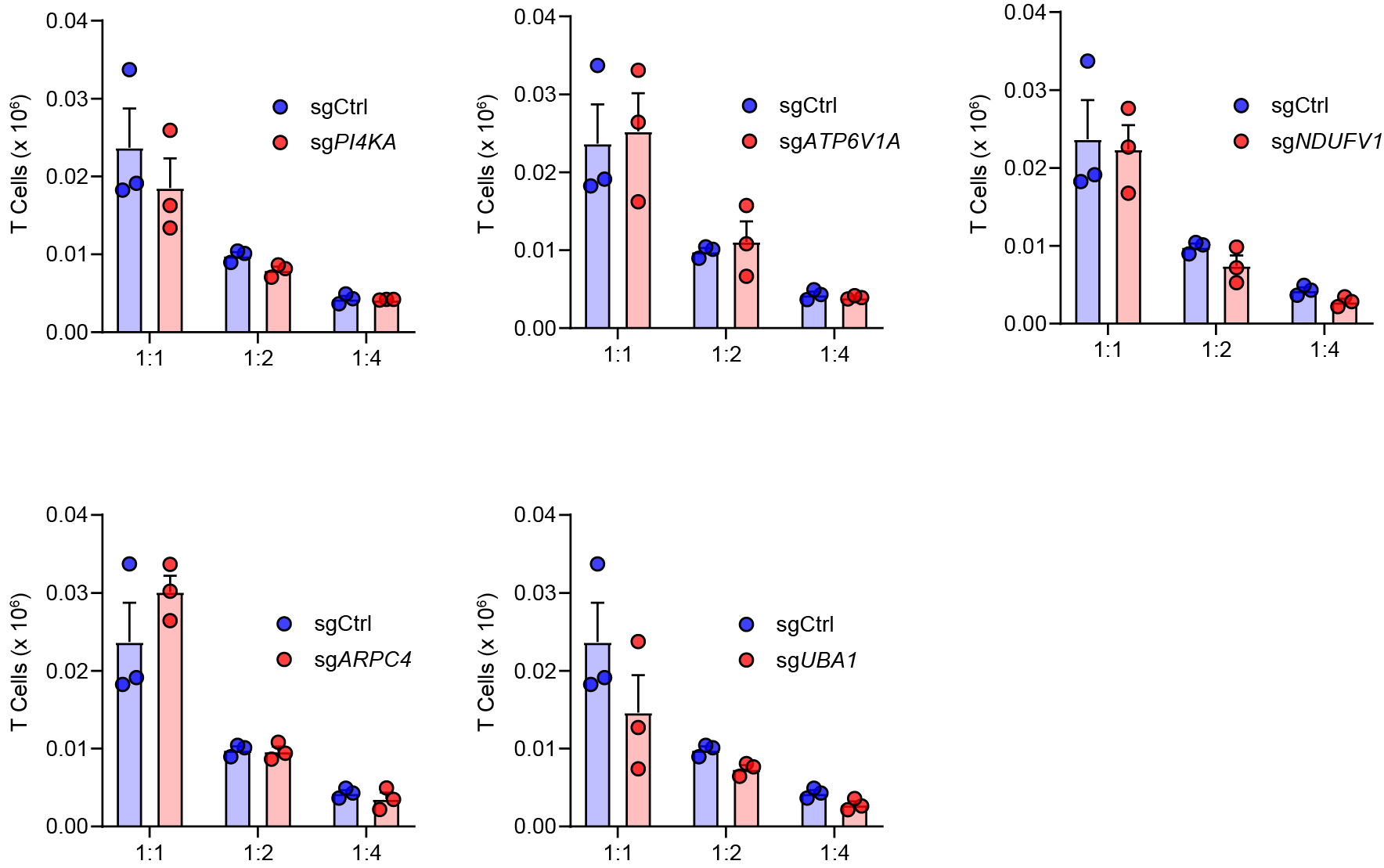


**Supplementary Figure 3. CAR T cell numbers after co-culture**

Quantifications of CAR T cell numbers by flow cytometry after 2-day co-culture with CRISPRi-U87 cells expressing the indicating sgRNAs at E: T ratios of 1:1, 1:2 and 1:4


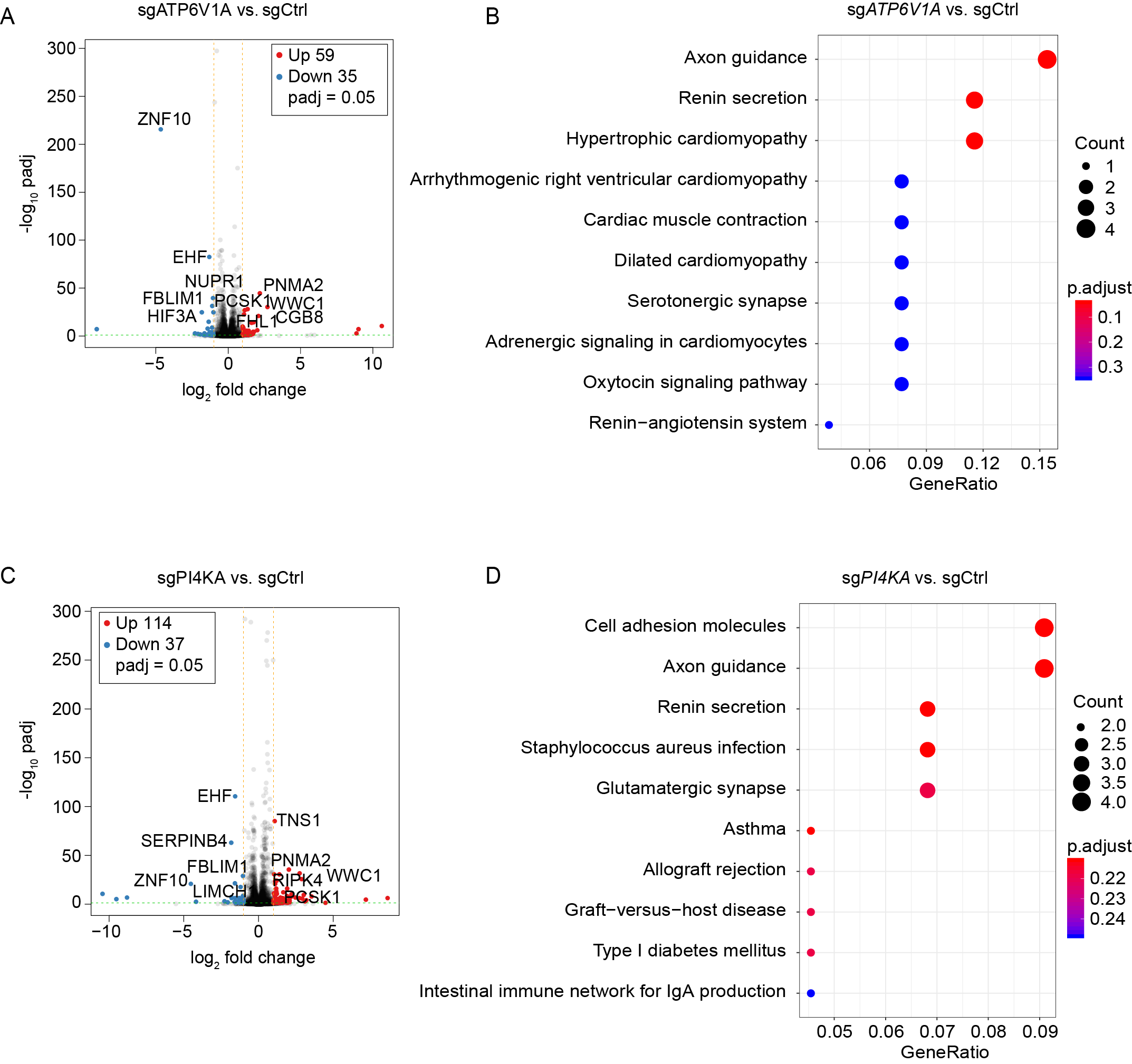


**Supplementary Figure 4. Transcriptome analysis on *ATP6V1A* and *PI4KA* knockdown cells**

**A & B.** Differentially expressed genes (**A**) and their enriched KEGG pathways (**B**) in *ATP6V1A* knockdown cells.

**C & D.** Differentially expressed genes (**C**) and their enriched KEGG pathways (**D**) in *PI4KA* knockdown cells.


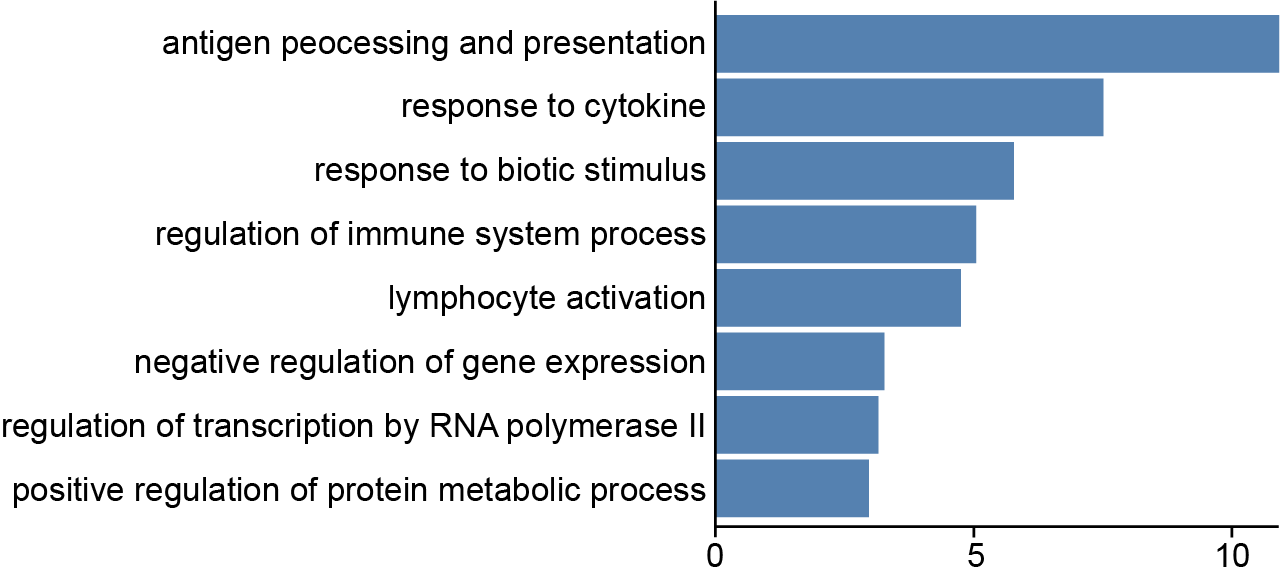


**Supplementary Figure 5. GO enrichment analysis for top 50 upregulated genes in CD8+ T cell following TL1A cytokine treatment *in vivo***
